# A Novel Synthetic Model of the Glucose-Insulin System for Patient-Wise Inference of Physiological Parameters From Small-Size OGTT Data

**DOI:** 10.3389/fbioe.2020.00195

**Published:** 2020-03-13

**Authors:** Sebastián Contreras, David Medina-Ortiz, Carlos Conca, Álvaro Olivera-Nappa

**Affiliations:** ^1^Centre for Biotechnology and Bioengineering (CeBiB), University of Chile, Santiago, Chile; ^2^Department of Chemical Engineering, Biotechnology and Materials, Faculty of Physical and Mathematical Sciences, University of Chile, Santiago, Chile

**Keywords:** mathematical modeling, glucose-insulin control, OGTT, inverse problems, personalized medicine

## Abstract

Existing mathematical models for the glucose-insulin (G-I) dynamics often involve variables that are not susceptible to direct measurement. Standard clinical tests for measuring G-I levels for diagnosing potential diseases are simple and relatively cheap, but seldom give enough information to allow the identification of model parameters within the range in which they have a biological meaning, thus generating a gap between mathematical modeling and any possible physiological explanation or clinical interpretation. In the present work, we present a synthetic mathematical model to represent the G-I dynamics in an Oral Glucose Tolerance Test (OGTT), which involves for the first time for OGTT-related models, Delay Differential Equations. Our model can represent the radically different behaviors observed in a studied cohort of 407 normoglycemic patients (the largest analyzed so far in parameter fitting experiments), all masked under the current threshold-based normality criteria. We also propose a novel approach to solve the parameter fitting inverse problem, involving the clustering of different G-I profiles, a simulation-based exploration of the feasible set, and the construction of an information function which reshapes it, based on the clinical records, experimental uncertainties, and physiological criteria. This method allowed an individual-wise recognition of the parameters of our model using small size OGTT data (5 measurements) directly, without modifying the routine procedures or requiring particular clinical setups. Therefore, our methodology can be easily applied to gain parametric insights to complement the existing tools for the diagnosis of G-I dysregulations. We tested the parameter stability and sensitivity for individual subjects, and an empirical relationship between such indexes and curve shapes was spotted. Since different G-I profiles, under the light of our model, are related to different physiological mechanisms, the present method offers a tool for personally-oriented diagnosis and treatment and to better define new health criteria.

## Introduction

Disequilibriums in the glucose-insulin (G-I) dynamics, as in diabetes, insulin resistance, glucose intolerance, among others, are a widespread condition in modern society (Cobelli et al., 2009; Ajmera et al., 2013; Hu et al., 2015; Toniolo et al., 2018). For this reason, the mathematical modeling of the G-I control system has been frequently visited, as shown by the wide variety of models presented in numerous reviews published to date (Bergman, 2005; Boutayeb and Chetouani, 2006; Makroglou et al., 2006; Palumbo et al., 2013; Cobelli et al., 2014).

For modeling purposes, we may understand the G-I dynamics as follows. The digestion of macronutrients generates glucose (among others nutrients), which enterocytes absorb into the bloodstream in the upper intestinal tract. The blood glucose concentration increase caused by glucose absorption induces pancreatic β-cells to secrete insulin in different timescales: early insulin release, signaled by the incretin hormones (secreted from intestinal enterocytes), is achieved by emptying the contents of the vacuoles in the β-cells. After that source is depleted, insulin production follows a saturable dynamics. Insulin signals the uptake of glucose from peripheral tissues (mainly muscle and adipose tissue), which metabolize it to obtain energy or to synthesize storage macromolecules, and the decrease of glucose release from the liver. If blood glucose levels are too low, pancreatic glucagon-induced hepatic glucose release restores the steady-state homeostatic level.

Some typical routine tests employed for the diagnosis of G-I-related dysregulations are the Oral Glucose Tolerance Test (OGTT) and the Meal Test (MT), which are widely used given their clinical simplicity and low cost. In OGTT, a fasting patient ingests a 75 g-controlled dose of liquid glucose (Bartoli et al., 2011), while in MT a controlled meal with known glycemic index (such as rice) is given to a patient. In both tests, glycemia is measured at different times. Typically, such measurements take place at time 0 (fasting) and 2 h after the ingestion of glucose, but more temporal resolution might be required, or other physiological variables measured, depending on how strict clinical criteria are. Moreover, efforts have been made to modify and standardize the temporal resolution and duration of the tests (Bergman et al., 2018). Besides the clinical interpretation of the OGTT and MT values, some model-derived indexes can be obtained, as is the case of the Insulin Sensitivity *S*_*I*_, derived from the very well known Minimal Model (Bergman et al., 1979). The insulin sensitivity *S*_*I*_ quantifies the ability of insulin to increase the effect of glucose on its own disappearance in a steady state. From Bergman's Minimal Model, mathematical models of the G-I dynamics have evolved, increasing their complexity and aiming to different objectives.

Stumvoll et al. (2000) presented an empirical approach based on correlations to determine *S*_*I*_ from OGTT curves. Mari et al. (2001) proposed a parametric approach to obtain this index, studying a population of 104 individuals with different clinical classification. High correlations between results for *S*_*I*_ calculated from the model vs. direct measurement for the full patient sample suggested the applicability of the OGTT to obtain clinically relevant parameters and perform large scale studies. Caumo et al. (2000) and Dalla Man et al. (2002, 2004, 2006) focused their efforts on modeling glucose absorption from the digestive system into the bloodstream, making differences between OGTT and MT. However, parametric identifiability in their models required the *a priori* knowledge of average values of some parameters of the sample, assumed equal and constant for all individuals. The number of patients (88) in Dalla Man et al. (2004) allowed to determine the non-Gaussian distributions of some of the parameters. Later, Dalla Man et al. (2007) presented a nested sub-systems model, fitting its parameters to a population of 204 clinically healthy individuals that underwent a MT. Reflecting on its complexity, the authors suggested to use this model only as a simulator. Following the trend of previous minimal models, this model also leaves out equations for other regulatory hormones such as glucagon, epinephrine, growth hormone, and incretins, which regain importance in other works (Brubaker et al., 2007; Silber et al., 2010; Mari et al., 2013).

Salinari et al. (2011) presented a model in which the intestinal absorption of glucose is obtained as a solution of a transport partial differential equation, where glucose is progressively absorbed while passing through the intestine, and stomach emptying is assumed to be exponential. Subsequently, De Gaetano et al. (2013) presented an extension of the classic minimal model, modeling the gastrointestinal tract as four compartments, coupled with first-order kinetics. Besides, the authors proposed fixed forms for hepatic glucose production and incretin action, without clear physiological justification or supporting background, and in disagreement with the state of the art (Silber et al., 2010). The patient sample analyzed in De Gaetano et al. (2013) comprised 78 patients with different clinical classifications, and parameters were fitted to whole groups of patients according to each clinical criterion, reporting statistical differences for insulin sensitivity between different groups.

The importance of the development of mathematical models for the study of the G-I control system lies in the potential to serve as support for clinical diagnostic tools in the detection of type II diabetes, insulin resistance, glucose intolerance, among other dysregulations of the G-I control system. However, at present, these models do not manage to effectively capture the differences that exist between patients in the way of achieving glycemic control (reflected as morphological variations in OGTT response curves), and do not represent it as a difference in the involved parameters that can be interpreted according to clinical criteria. Additionally, the more complex models mentioned above have not been used to infer physiological parameters for an individual patient from an OGTT, since they have only been used to calculate average parameters in a group of patients, or required special clinical setups to obtain the data needed for fitting them. This is partly due to the intrinsic complexity of the models and the number of parameters involved, but also to the lack of a numerical procedure for parameter fitting. Therefore, a mathematical model capable of accounting for different physiological states and applicable as a tool for clinical diagnosis becomes necessary for personalized medicine.

In the present work, we present a synthetic mathematical model to represent the G-I dynamics in OGTT using Delay Differential Equations (DDE), in which each parameter describes a single physiological phenomenon. To the best knowledge of the authors, this is the first DDE model involved in describing OGTT dynamics, including the mutual interrelation between glucose and insulin. The structure of the model allowed for representation of every observed qualitative dynamic behavior in our cohort, regardless of the number, height or location of the glucose and insulin peaks. Using a novel information-based approach we achieved an individual-wise parameter fitting using the five glycemia and insulinemia points of a routine OGTT directly, for a cohort of 407 patients that underwent a 75-g OGTT, which is the largest cohort analyzed so far for this end. We also show that there are different controlling behaviors within the clinical normality thresholds, accounting for different physiological mechanisms to achieve glycemic control. Given that parameter fitting is individual, it would be possible to classify each patient within different groups, suggesting that different dysregulated mechanisms require different corrections, thus transforming the proposed model and fitting procedure into a tool for preventive clinical diagnosis and personalized medicine.

## Materials and Methods

A cohort of 407 volunteers was used for testing the capabilities of our model and our patient-wise parameter recognition methodology. All volunteers gave their informed consent to use their OGTT data in this study. All volunteers in this sample were clinically healthy according to criteria used in Chile, in strict clinical settings to characterize normoglycemic patients. According to these criteria, a patient who has basal (stable overnight) glycemia lower than 100 mg/dL, basal insulinemia lower than 15 μU/mL, glycemia values not exceeding 160 mg/dL at any time and not persisting at values higher than 140 mg/dL over 2 h, and insulinemia not persisting at values higher than 60 μU/mL for a continuous time period of 120 min, would be classified as normal. Approximately 80% of the cohort corresponded to female patients, with ages ranging between 18 and 65 years-old. Nevertheless, no statistical sex-related difference was found within the cohort. Every patient underwent an OGTT with five measurements for both glycemia and insulinemia: fasting (basal state) and every 30 min, for 2 h. Further description of the data, as statistical properties, histograms of the measurements at every time and of the age distribution are presented in Supplementary Material (Table S1 and Figures S1, S2, respectively).

**Figure 1:**
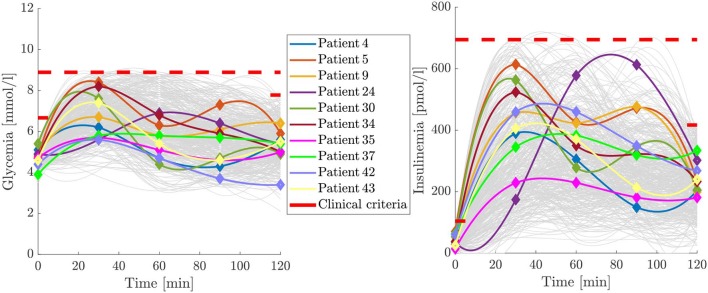
Some of the different G-I profiles encompassed under the clinical normality criterion. Continuous curves are spline interpolations of the diamond marked experimental trends. Note that similar glycemia curves are not necessarily associated with similar insulinemia curves, and a single-peak profile in glycemia is not necessarily associated with a single-peak insulinemia profile. Upper clinical normality criteria are represented as red limits for the basal and last points and for the entire OGTT time span.

We identified many radically different G-I profiles among the population. Examples of them are hypoglycemic individuals, single/double peak patients, and those with practically invariant G-I profiles, as shown in Figure 1. In Figure 1, the solid lines correspond to a spline interpolation of the experimental measurements, which are marked with solid diamonds of the same color. These different OGTT profiles could account for diverse physiological states related to gastric emptying, intestinal absorption, or other components of the glycemic control system, masking pre-disease conditions under the concept of normality. Therefore, as currently defined, to identify the physiological background behind a clinically healthy or unhealthy individual seems to be an ill-posed inverse problem.

The implementation of the mathematical model and resolution of the parameter-fitting inverse problem was performed in Matlab version R2017a, using the Global Optimization Toolbox. All scripts and routines for parameter fitting were run on the Chilean National Laboratory for High-Performance Computation (NLHPC) servers, using BASH-based control scripts.

## Results

### Synthetic OGTT Glycemia-Insulinemia Model

**Figure 2:**
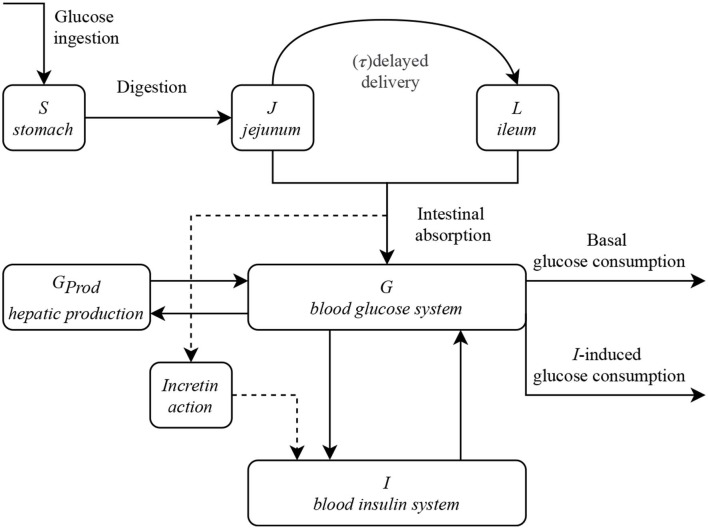
Box diagram of the proposed model and the interactions between the different compartments and variables. The gastrointestinal tract, namely variables *S, J*, and *L*, are decoupled from the blood G-I dynamics.

The synthetic model proposed in this work considers five main variables. Four of them represent the amount or concentration of glucose in different compartments: in the stomach *S*, in the upper intestinal tract *J* (jejunum) and *L* (ileum), and in the bloodstream (glycemia) *G*. The last variable accounts for the insulinemia *I*. These different variables interact as illustrated in the box diagram of Figure 2, in ways that will be further detailed in this section. We follow the notation of De Gaetano et al. (2013), but our model also considers the contributions of other works (Dalla Man et al., 2007; Salinari et al., 2011; De Gaetano et al., 2013), together with our own developments.

Figure 2 shows the interdependence of the different variables of the model. The gastrointestinal sections are decoupled from the blood G-I dynamics, which, as we will see, are connected in a very non-linear fashion. To obtain the G-I profiles of a particular individual, given all the parameters involved, it is necessary to solve the system of differential equations that we propose to model it. All the proposed constitutive equations may be found in this section, together with their physiological background and the reasons behind its mathematical formulation. A summary of the model is presented in the last subsection and a detailed list of its parameters and variables can be found in Tables S2, S3.

#### Gastrointestinal System

To mathematically represent the stomach emptying for a liquid bolus, we assume its rate $v_{s}=\frac{dS}{dt}$ to be directly proportional to the content of glucose at every time, *S*(*t*), as Equation (1) shows:

(1) $$\begin{array}{l} \frac{dS}{dt}=-k_{js}S,\;S\left(0\right)=D, \end{array}$$

where *k*_*js*_ is a first-order kinetic constant, *D* the ingested glucose bolus in an OGTT (75 g), and the minus sign accounts for the disappearance of glucose, hence the emptying of the stomach. The glucose that leaves the stomach appears in the jejunum *J*, being a source term in the rate (Equation 11).

As absorption of glucose may take place in this section of the small intestine, we may close the mass balance noting that the glucose that is not absorbed will continue its way, being transported to the ileum *L*.

(2) $$\begin{array}{l} \frac{dJ}{dt}=\underset{\text{Stomach delivery}}{\underset{︸}{k_{js}S}}-\underset{\text{Absorption into the bloodstream}}{\underset{︸}{k_{gj}J}}-\underset{\text{Delivery to }L}{\underset{︸}{k_{jl}J}},\;J\left(0\right)=0 \end{array}$$

The structure of our model, from this point and further, is significantly different from the one presented in De Gaetano et al. (2013). De Gaetano et al. (2013) suggested that to represent the effect of intestinal transit between jejunum and ileum, a virtual compartment *R* can be added in between, where absorption does not occur. Nevertheless, the equations proposed to reach such objective are not entirely appropriate for representing different curve shapes, especially those curves with delayed 2-peak dynamics with equal peak height and width or a higher second peak (see Figure 1).

Here we decided to follow the formalism presented by Salinari et al. (2011) to represent intestinal transit. Taking into account the peristalsis-driven intestinal transit mechanism, we can assume there is no mixing in the axial axis. Hence, we model the flow through the intestine as a plug-flow reactor with uniform velocity *U*. To represent the distribution of glucose transporters along the small intestine, we assume that absorption occurs in two separated areas of the intestine, the jejunum and the ileum, located at a distance *l*, so the time it takes for the ileum to receive glucose transiting from the jejunum is τ := *l*/*U*. Consequently, instead of having a spatial partial differential equation for intestinal absorption, we have two ordinary differential equations, one for the jejunum (Equation 11) and one for the ileum, the last one having a τ−delayed forcing function (Equation 3).

(3) $$\begin{array}{l} \frac{dL}{dt}=\underset{\text{Delayed contribution from }J}{\underset{︸}{k_{jl}\varphi \left(t\right)}}-\underset{\text{Absorption into the bloodstream}}{\underset{︸}{k_{gl}L\left(t\right)}},\;\varphi \left(t\right)=\{\begin{array}{l} 0,\text{ if }t<\tau \\ J\left(t-\tau \right),\text{if }t\geq \tau \end{array} \end{array}$$

where *k*_*jl*_ and *k*_*gl*_ are first-order kinetic constants, respectively, accounting for the rate of jejunal glucose delivery and the glucose absorption into the bloodstream.

#### Blood Glucose Dynamics

To represent variations in glycemia, our model contemplates the following control mechanisms. As source terms in the glycemia equation, we considered the intestinal absorption of glucose adjusted by glucose bioavailability (η), following the form presented in Dalla Man et al. (2002), and the hepatic contribution to glucose homeostasis (*G*_prod_), which indirectly accounts for the action of glucagon. The sink terms in the equation represent glucose uptake by insulin-insensitive tissues and renal excretion, which is proportional to *G*, and insulin-driven consumption of glucose, taking place in insulin-sensitive tissues, which is proportional to *GI*. Equation (4) represents mathematically the glycemia dynamics.

(4) $$\begin{array}{l} \frac{dG}{dt}=\underset{\text{Basal uptake}}{\underset{︸}{-k_{xg}G}}-\underset{\text{Insulin-sensitive uptake}}{\underset{︸}{k_{xgi}GI}}+\underset{\text{Hepatic delivery}}{\underset{︸}{G_{\text{prod}}}}+\underset{\text{Intestinal absorption}}{\underset{︸}{\eta \left(k_{gj}J+k_{gl}L\right)}},\;G\left(0\right)=G_{b} \end{array}$$

where *k*_*xg*_ is the insulin-independent glucose uptake rate, *k*_*xgi*_ is the uptake rate of insulin-sensitive tissues, η is the bioavailability of the intestinal absorbed glucose, and *G*_prod_ is the rate of hepatic glucose production.

#### Hepatic Glucose Production Function

We implicitly incorporated the effect of glucagon into a hepatic glucose production function, which is an always positive term that contributes to the equation of blood glucose dynamics. Previous works model this contribution with exponential functions (De Gaetano et al., 2013; Erlandsen et al., 2018), without a solid physiological background but rather a mathematical convenience. However, when analyzing the functional nature of such expressions, we realize that the differential mechanism is not entirely clear since the function derivative cannot be written as a function of itself. Therefore, no reliable physiological mechanism supports the form of such rate functions. We propose *G*_prod_ as the complement of a Monod-like equation, which is typically used to model problems of saturable growth, production, enzymatic reaction and receptor/ligand interaction or transport:

(5) $$\begin{array}{l} G_{\text{prod}}=\frac{k_{\lambda }}{k_{2}+G}, \end{array}$$

As defined above, *G*_prod_ is also the solution of the mechanism given by Equation (6), which represents a logistic hyperbolic production with an asymptotic maximum rate (see derivation in Supplementary Material).

(6) $$\begin{array}{l} \frac{dG_{\text{prod}}}{dG}=-\frac{G_{\text{prod}}}{G}\left(1-\frac{k_{2}}{k_{\lambda }}G_{\text{prod}}\right), \end{array}$$

By imposing steady-state conditions $G_{\text{prod}}\left(G_{b}\right)=G_{\text{prod}}^{0}$, *k*_2_ can be written as a function of the other variables, resulting in Equation (7), i.e., a saturable Michaelis-Menten-like kinetics much more representative of the physiological background of cellular processes. Noticeably, even though the rate Equation (6) does not include any set point, the integrated steady-state hepatic glucose production (Equation 7) explicitly depends on the difference between glycemia and its base level (see derivation in Supplementary Material).

(7) $$\begin{array}{l} G_{\text{prod}}=\frac{k_{\lambda }}{\frac{k_{\lambda }}{G_{\text{prod}}^{0}}+\left(G-G_{b}\right)}. \end{array}$$

#### Blood-Insulin Dynamics

For the blood-insulin system, following the model in De Gaetano et al. (2013), we propose a Hill's dynamics for pancreatic secretion (Goutelle et al., 2008), and an *I*-proportional degradation term. Noteworthy, these dynamics exhibit a saturation behavior since it is formulated exclusively for OGTT circumstances. However, we corrected the mathematical form of the incretin action suggested in De Gaetano et al. (2013). De Gaetano et al. (2013) assumed that incretin secretion is proportional to glucose levels within the intestinal lumen. Nonetheless, it has been shown that it rather depends on the rate of absorption of glucose from the intestinal lumen (Silber et al., 2010). Therefore, we corrected this in Equation (8):

(8) $$\begin{array}{l} \tilde{G}=G+f_{gj}\left(k_{gj}J+k_{gl}L\right). \end{array}$$

Equation (8) makes sense from a physiological point of view since intestinal epithelial cells are not able to sense the absolute amount of glucose in the intestine due to the lack of glucose sensor proteins, but their internal metabolic rates are directly dependent on the steady-state cytoplasmic concentration of glucose, which is proportional to glucose membrane transport through the cell. In this equation, *f*_*gj*_ is a conversion factor that indirectly links glucose absorption rate to insulin secretion rate through incretin action, thus representing the relative power of incretin action vs. direct glycemic action on the pancreas. Under these assumptions, Equation (9) gives the final expression for the insulin dynamics:

(9) $$\begin{array}{l} \frac{dI}{dt}=k_{xi}I_{b}\left(\frac{\beta^{\gamma }+1}{\beta^{\gamma }{\left(\frac{G_{b}}{\tilde{G}}\right)}^{\gamma }+1}-\frac{I}{I_{b}}\right) \end{array}$$

where *k*_*xi*_ is a first-order kinetic constant for the insulin degradation in target tissues, β and γ are parameters for half saturation and acceleration of the insulin production, which account for first and second phase pancreatic secretion, $\tilde{G}$ the apparent *G*, enhanced by incretin action, and *G*_*b*_, *I*_*b*_ the steady-state value of such variables.

#### Insulin Action on Glycemia and Insulin Sensitivity

Former mathematical models including insulin action were divided in models that considered a direct action of blood insulin on tissues to regulate glucose uptake and more complex models that considered an additional intermediate compartment. Such compartment represented the interstitial fluid in peripheral tissues, into which insulin was absorbed from the bloodstream following a first-order kinetics, and only then could exert its action. Mathematically, the effect of this formulation in the more complex models was the introduction of a small delay and a proportionality constant between concentrations in the bloodstream and the interstitial fluid, which caused a small decrease in peak height and slight broadening of peak width for insulin in the intermediate compartment compared to the bloodstream. Application of these models to experimental data demonstrated a minimal delay, in the range of a few minutes, between concentrations in the bloodstream and the intermediate active compartment. Taking into account that usual OGTT experiments take measures every 30 min and the registered G-I dynamics occur in the order of hours, this small delay was not included in the formulation of our model, because the time resolution might result too coarse to accurately calibrate such parameters. In this way, we only consider a direct action of blood insulin on target tissues. From a mathematical and practical point of view, this decision also resulted in a more compact model with fewer parameters to fit experimental data.

Insulin sensitivity *S*_*I*_, formally introduced by Bergman et al. (1979) and mathematically defined by Equation (10), accounts for the quantitative influence of insulin to increase the effect of glucose on its own disappearance, in steady state.

(10) $$\begin{array}{l} S_{I}=\frac{\partial E}{\partial I},\;E=-\frac{\partial \left(dG/dt\right)}{\partial G} \end{array}$$

In our mathematical model, we adopted the term *k*_*xgi*_*GI* and *k*_*xg*_*G* of De Gaetano et al. (2013) to represent the glucose-mediated effect of insulin on glucose disappearance from the bloodstream and glucose uptake by insulin-independent tissues, respectively. However, contrary to the equations of De Gaetano et al. (2013), in our model parameter *k*_*xgi*_ is a true insulin sensitivity value. This was achieved by the redefinition of *G*_prod_, resulting in such a way that no additional terms appeared in the mathematical calculation of the insulin sensitivity according to Equation (10).

#### Summary of the Model

Collecting the different expressions derived in the previous sections for the constitutive compartments of our model, we can summarize it in the following system of differential equations:

$$\begin{array}{l} \frac{dS}{dt}=-k_{js}S,\;S\left(0\right)=D\\ \frac{dJ}{dt}=k_{js}S-k_{gj}J-k_{jl}J,\;J\left(0\right)=0\\ \frac{dL}{dt}=k_{jl}\varphi \left(t\right)-k_{gl}L\left(t\right),\;\varphi \left(t\right)=\{\begin{array}{l} 0,\text{ if }t<\tau \\ J\left(t-\tau \right),\text{if }t\geq \tau \end{array}\\ \frac{dG}{dt}=-\left(k_{xg}+k_{xgi}I\right)G+G_{\text{prod}}+\eta \left(k_{gj}J+k_{gl}L\right),\;G\left(0\right)=G_{b}\\ \frac{dI}{dt}=k_{xi}I_{b}\left(\frac{\beta^{\gamma }+1}{\beta^{\gamma }{\left(\frac{G_{b}}{\tilde{G}}\right)}^{\gamma }+1}-\frac{I}{I_{b}}\right) \end{array}$$

### Parameter Fitting Strategy

During a 5-point OGTT, five experimental measures of both glucose and insulin are captured, generating vectors *G*_exp_ and *I*_exp_. By protocol, measurements are taken at 0, 30, 60, 90, and 120 min after glucose ingestion, giving a time vector *T*_exp_ = [0 30 60 90 120]. The model detailed in the previous sections was used to represent and interpolate these points continuously. Let $G_{\text{num}}\left(\vec{\theta }\right),I_{\text{num}}\left(\vec{\theta }\right)$ be its solution for the G-I dynamics, with parameters $\vec{\theta }$. The traditional way of formulating the parametric fitting problem is by minimization of a cost function *J*_exp_ that accounts for the difference between the modeled curve and experimental measurements. Generally, this function is proportional to the mean squared error *MSE*,

(11) $$\begin{array}{l} J_{\text{exp}}\left(\vec{\theta },\alpha \right)=\frac{1}{5{\left(G_{\text{max}}-G_{\text{min}}\right)}^{2}}\overset{5}{\underset{i=1}{\sum }}{\left(G_{\text{exp}}\left(i\right)-G_{\text{num}}\left(T_{i}\right)\right)}^{2}\\ \;+\frac{\alpha }{5{\left(I_{\text{max}}-I_{\text{min}}\right)}^{2}}\overset{5}{\underset{i=1}{\sum }}{\left(I_{\text{exp}}\left(i\right)-I_{\text{num}}\left(T_{i}\right)\right)}^{2}, \end{array}$$

where α is a constant that connects the contribution of the insulin curve to *MSE* and ${\left(G_{\text{max}}-G_{\text{min}}\right)}^{2}$ is a scaling factor. This problem consists of finding the values of all 13 parameters of the model from 10 experimental measures obtained during a routine 5-point OGTT for a given patient, which is a slightly underdetermined problem. However, we can exploit the knowledge we have about the nature of the physiological G-I response, accumulated in more than 40 years of routine testing and modeling, to gain in robustness and identifiability of the parameter set for each patient. We identified and used the following strategies for improvement:

- Increasing data density through the use of interpolators, to favor smoothness and regularity of the solutions, and to penalize nonphysiological oscillations.
- Simulation-based regularization for clusters of similar curves.
- Nested sub-problems and sequential approximations to build a robust initial guess.
- Incorporation of information in the cost function and the delimitation of the feasible set.
- Algorithm choice for the parameter recognition problem and final shaping of the feasible set.

#### Increasing Data Density

Given the nature of the equations presented in our model, non-physiological high-frequency oscillatory solutions might appear. Taking into account (a) the nature of the physiological G-I control system, (b) the oscillations measured experimentally in the literature, and (c) the 30-min apart measurements taken during a 5-point OGTT, we know that high-frequency oscillations –relative to the sampling time– should not be observable in our solution. Therefore, we propose to favor those solutions that only have low-frequency oscillations, somehow forcing the it to resemble the experimental data in a smooth way. For this, we propose to increase the density of putative measured points using a soft interpolant to connect the experimental measurement points. Without loss of generality, for *G*, the interpolant *Ĝ* used to increase the data density is defined by Equation (12):

(12) $$\begin{array}{l} Ĝ=\phi G_{\text{spline}}+\left(1-\phi \right)G_{\text{pol}}, \end{array}$$

that is a convex combination between a cubic spline and a low-degree polynomial interpolator. We can define a new component of the error function, *J*_spline_, based on the *MSE* between *Ĝ* and *G*_num_, following the structure of Equation (11). It is important to point out that this contribution has no greater effect than favoring those solutions that are smooth and regular. Introducing this component into the curve fitting procedure adds information because the optimizer would not only look for those solutions whose numerical profiles match the experimental data points, but for those whose profiles also do not drift considerably from the expected trend.

#### Simulation-Based Regularization for Clusters of Similar Curves

Taking into account the physiological and molecular mechanisms involved in the G-I dynamics and the experimental values obtained in typical OGTT measurements, we determined plausible lower and upper bounds (θ_*i*,min_ and θ_*i*,max_, respectively) for each parameter θ_*i*_. In a first stage, we may define the feasible set *F*_0_ considering such thresholds,

(13) $$\begin{array}{l} F_{0}=\overset{13}{\underset{i=1}{⊗}}\left[\theta_{i,\text{min}},\theta_{i,\text{max}}\right], \end{array}$$

where the operator ⊗ represents the Cartesian product between the intervals defined by the lower and upper thresholds of each parameter. We performed a simulation stage to explore the nature of *F*_0_, in which we simulated 10^8^ values of $\vec{\theta^{*}}\in F_{0}$, and studied the $G_{\text{num}}\left(\vec{\theta^{*}}\right)$ and $I_{\text{num}}\left(\vec{\theta^{*}}\right)$ profiles obtained. If such profiles fulfilled the clinical normality criteria, they were assigned to groups of experimental profiles based on their similarities, aiming to build a set of initial guesses for the parameters corresponding to such individuals. Once we had enough $\vec{\theta^{*}}$ for each group of curves, a fourth component for the global cost function (Equation 16) was added, accounting for the contribution of local regularization near $\vec{\theta_{j}^{*}}$ characteristic of the *j*'th group.

#### Nested Sub-problems and Sequential Approximations

We followed a staged approach for the construction of initial guesses for the inverse problem, summarized in the following algorithm:

**Algorithm 1:**
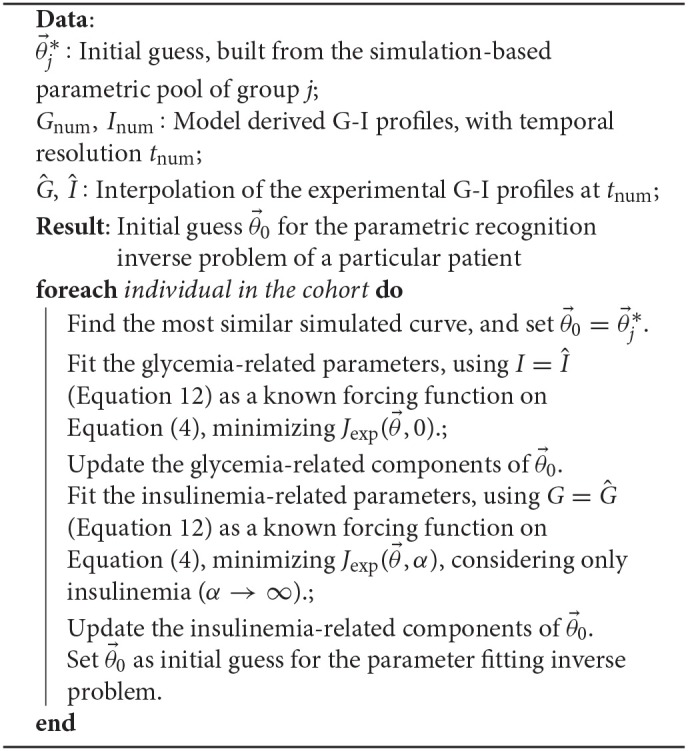
Sequential approximation to the initial guess

Steps one and two aim to build an appropriate initial guess for the parameter fitting problem, solved in step three. This step does not include further assumptions on the nature of the solutions, but only the model equations.

#### Incorporation of Information in the Cost Function

Given that the *a priori* identifiability of the model is not guaranteed (De Gaetano et al., 2013), we corrected the cost function presented in the previous section to incorporate more information. Without making any assumption concerning the parameters, we incorporated information from clinical records and from the test itself. In particular. we added terms that account for: (i) experimental errors, associated with sampling time and laboratory techniques, and (ii) expected extreme values (maxima and minima), inferred from the experimental measurements and based on clinical criteria.

Regarding the first term, associated with experimental errors in both time and measurement, such 2− D variability transformed each data point into an ellipse in the (*t, G*) or (*t, I*) space, centered on the experimentally determined value *G*_exp_ or *I*_exp_, at time *T*_exp_. We used a constant Δ_*t*_ = ±3 min as a scale for the temporal uncertainty (horizontal semi-axis of the ellipse), meanwhile a proportional contribution (to the *G*_exp_ or *I*_exp_ values) was chosen for the vertical axis. The weight of this error source in the total cost function was calculated based on a polar probability density of ρ(*r*, θ), as described in algorithm 2. Note that when considering together *J*_exp_ and *J*_error_, the algorithm can be significantly simplified, as the calculations for the case *d* = 0 may be skipped only giving a greater weight to the contribution of *J*_exp_.

**Algorithm 2:**
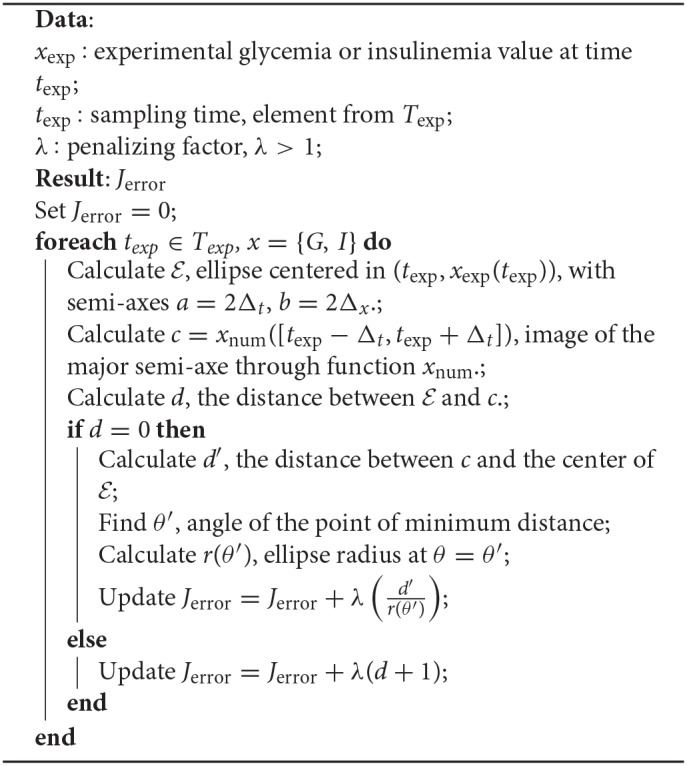
Incorporation of experimental errors in the cost function

Regarding the second term, its derivation was—in a mathematical sense—more complicated. Given the characteristics of the 5-point OGTT considered on this work, it seems reasonable to expect that the maximum value of the modeled glycemia curve should be of the same order of the maximum of the experimental profile. Consequently, we will have an idea of the time $t^{*}\sim T_{\text{exp}}\left(i\right)$ when our model reaches such value. Furthermore, considering a sequence of experimental measurements *H*_*i*_ recorded at times *T*_*i*_, which approximate a $C^{1}$ function *h*(*t*). If for certain *i*_0_ it is fulfilled that

(14) $$\begin{array}{l} \left(H_{i_{0}}-H_{i_{0}-1}\right)\left(H_{i_{0}+1}-H_{i_{0}}\right)<0, \end{array}$$

there exists a time $t^{*}\in \left[T_{i_{0}-1},T_{i_{0}+1}\right]$ where *h*′(t^*^) = 0. Note that this can happen more than once for a sequence of experimental measurements so that we may have more equations of the form $h^{\prime }\left(t_{j}^{*}\right)=0$. For our case, as the OGTT data consists of five glucose and five insulin measurements, the maximum number of additional equations we can have is 6.

The classical way of solving the direct problem of finding maximum/minimum values of a function *h*(*t*) is finding a time t^*^ in which *h*′(t^*^) = 0 and a sign change occurs in the same point. Then, $h_{\text{opt}}=h\left(t^{*}\right)$. This same reasoning can be applied to glycemia, using Equation (4) and imposing $\frac{dG}{dt}=0$ to obtain Equation (15):

(15) $$\begin{array}{l} G^{\text{opt}}=\frac{G_{\text{prod}}^{*}+\eta \left(k_{gj}J^{*}+k_{gl}L^{*}\right)}{k_{xg}+k_{xgi}I^{*}}, \end{array}$$

where *G*^opt^ is the maximum glycemia, and $I^{*},G_{\text{prod}}^{*},J^{*},L^{*}$ are, respectively, the insulin concentration, hepatic glucose production rate, and the amount of glucose in the jejunum and ileum at time t^*^ (when *G*^opt^ is reached). Note that the formulation is general, and it serves for any point of derivative equal to zero (which we can estimate from the clinical exam trends). We can indeed follow and apply the same idea for the insulin equation (Equation 9). If we do this for all critical points we will have *n* linearly independent equations, since the times t^*^ and optimal values of *G, I* will be different. We can think of each equation as the *i*− th component of a function $F\left(\vec{\theta }\right)=\overset{n}{\underset{i=1}{\sum }}f_{i}{\vec{e}}_{i}$. Note that around $\vec{\theta^{*}}$ where $F\left(\vec{\theta^{*}}\right)=0$, the parameters can be expressed as implicit functions of the known variables (such as *G*_*i,max*_ and *I*_*i,max*_). These equations will define different loci for the fitted parameters. The problem that appears directly after this definition is that known variables, in reality, are only approximately known within a confidence interval *I*_*c,i*_. Then, when incorporating them, we will have manifolds (of loci). More specifically, we say that $\vec{\theta }$ satisfies the equation *i*, if there are $G_{i,max}^{*},I_{i,max}^{*},t^{*}\in I_{c,i}$ such that $f_{i}\left(\vec{\theta }\right)=\vec{0}$. We then build an information function, which conditions the shape of the feasible set, tagging as unfeasible the solutions $\vec{\theta }$ such that $F\left(\vec{\theta }\right)=\overset{n}{\underset{i=1}{\sum }}f_{i}\left(\vec{\theta }\right){\vec{e}}_{i}\neq \vec{0}.$ Thus, we will have a new feasible set for our solutions and a new approach to the problem.

#### Settings of the Inverse Problem: Functions, Algorithms, and Thresholds

Finally, including all the contributions mentioned above, the parameter-fitting problem can be formulated as:

(16) $$\begin{array}{l} \underset{\text{Information function}}{\underset{︸}{\underset{\vec{\theta }\in F_{0}|F\left(\vec{\theta }\right)=\vec{0}}{\min}}}\underset{\text{Experimental data}}{\underset{︸}{\lambda_{1}J_{\text{exp}}\left(\vec{\theta },\alpha \right)}}\;+\;\underset{\text{Interpolator}}{\underset{︸}{\lambda_{2}J_{\text{spline}}\left(\vec{\theta },\alpha \right)}}\;+\;\underset{\text{Experimental errors}}{\underset{︸}{\lambda_{3}J_{\text{error}}\left(\vec{\theta },\alpha \right)}}\;+\;\underset{\text{Local regularization}}{\underset{︸}{\epsilon ∥\vec{\theta }-\vec{\theta_{j}^{*}}∥}}, \end{array}$$

with ϵ arbitrarily small, and which solution is the set of parameters that characterize the glycemic-insulinemic control for each patient. The resolution of the minimization problem 16 at every stage was achieved by combining deterministic methods (gradient search) and heuristic methods such as simulated annealing and pattern search, available in the Matlab Global Optimization Toolbox. Parameters were obtained for the whole studied cohort.

## Discussion

### Goodness of Fit

Applying the parameter recognition procedure presented in previous sections, it was possible to fit our model to the experimental OGTT glycemia and insulinemia profiles of the whole studied cohort, thus obtaining the physiological parameters that control the observed trends for each patient.

**Figure 3:**
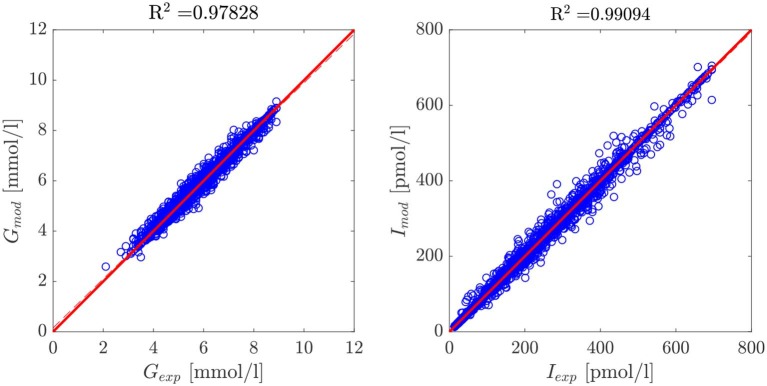
Scatter plot of model-predicted glycemia and insulinemia vs. experimental measurements for the entire cohort of 407 patients. The data cloud lies in the identity zone, without significant deviations from it, accounting for a good fit.

**Figure 4:**
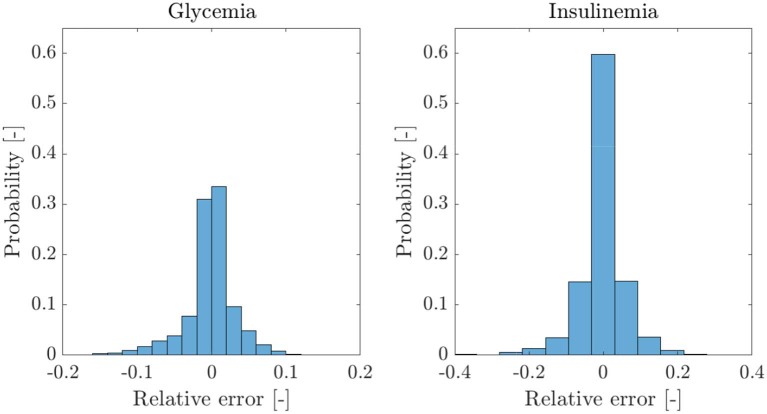
Probability histograms of relative errors of the model predictions for the cohort of 407 patients and five data points per patient. Given the Gaussian nature of the residues of both variables, we can safely discard the presence of bias.

To evaluate the performance of our model and the proposed parameter recognition procedure, we studied the quality of their predictions for both glycemia and insulinemia. Figure 3 shows the scatter plots of experimental vs. predicted values for glycemia and insulinemia for all 407 patients, where both predicted variables follow the expected trend. The data point cloud lies in the identity zone, without significant deviations, and the variables show high correlation. The probability-normalized residues histogram of both variables had a Gaussian nature, as depicted in Figure 4, with low variance. In sum, prediction errors are normally distributed and unbiased, while predictions are highly correlated to experimental measures, which demonstrates the goodness of fit of our model and method.

### Sensitivity Analysis

**Figure 5:**
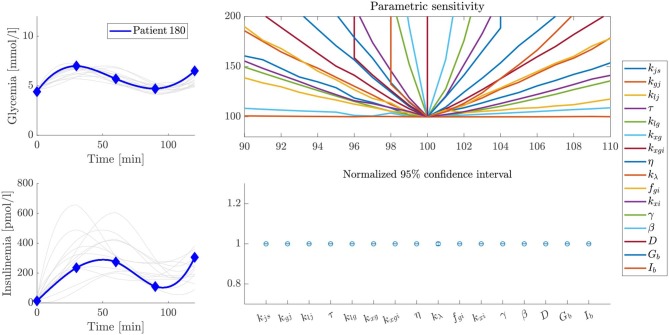
Sensitivity and stability analysis of fitted parameters for patient 180. The figure shows the glycemia and insulinemia profiles of the patient in the frame of patients that have similar glycemic curves (upper and lower left plots, respectively). A volcano plot, as described in the text, is presented in the upper right plot, and a representation of the width of the 95% confidence interval for each parameter is presented in the lower right plot. In this example, even though some parameters have a low slope over the studied interval, the variability of their values, expressed as the width of the 95% confidence interval, is almost negligible.

**Figure 6:**
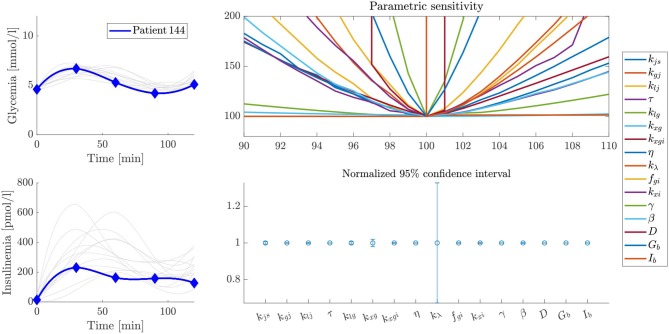
Sensitivity and stability analysis of fitted parameters for patient 144. The figure shows the glycemia and insulinemia profiles of the patient in the frame of patients that have similar glycemic curves (upper and lower left plots, respectively). A volcano plot, as described in the text, is presented in the upper right plot, and a representation of the width of the 95% confidence interval for each parameter is presented in the lower right plot. In this example, the variability of the *k*_λ_ parameter is considerably higher than in the case of Figure 5, probably because of differences in the experimental profiles.

**Figure 7:**
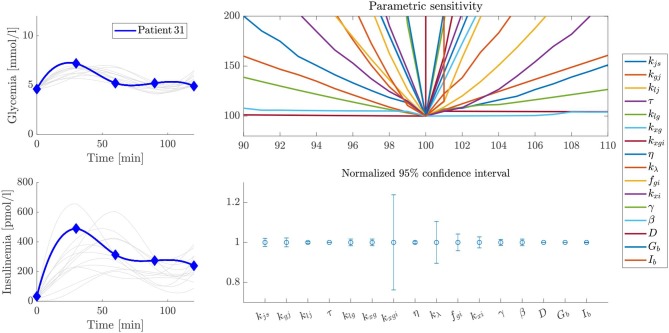
Sensitivity and stability analysis of fitted parameters for patient 31. The figure shows the glycemia and insulinemia profiles of the patient in the frame of patients that have similar glycemic curves (upper and lower left plots, respectively). A volcano plot, as described in the text, is presented in the upper right plot, and a representation of the width of the 95% confidence interval for each parameter is presented in the lower right plot. In this example, the variability of some parameter values is not negligible.

Parameter sensitivity was evaluated for each patient by analyzing how variations in parameter values affected the error between predicted and experimental values (the cost function defined by Equation 16). Some particular examples are shown by the volcano plots in Figures 5–7. In these figures, the greater the slope around the central point, the more sensitive the patient is to variations of that parameter. Therefore, a more sensitive parameter suggests higher confidence in its fitted value. We also performed ten different parameter fitting experiments for each patient, starting from different initial values and using the deterministic and heuristic procedures described above. With these results, we calculated a 95% confidence interval for the parameters of each patient, as shown in Figures 5–7. Following our former reasoning, all parameters with high slopes around the central point in the volcano plot have very narrow confidence intervals, but unexpectedly some parameters with low sensitivity in volcano plots have also very narrow confidence intervals in fitting experiments. This observation demonstrates that parameter accuracy following our fitting method is higher than expected from analyzing parameter sensitivity around a central point.

Analyzing differences between patients, our results show that this sensitivity analysis differs for each case, since different curve shapes suggest different physiological ways to achieve glycemic control. Hence, the relative importance of each parameter on the control mechanism, and the reliability of their values, can be considered proportional to their sensitivity or the extension of their confidence interval. As the most sensitive parameters are those that determine the shape of the control profile, understanding their individual and collective meaning may give valuable information about the health status of each patient, highlighting the physiological background of the observed trend.

We present some examples of these observations in Figures 5–7, which feature a special kind of graph that we will address as a volcano plot. In a volcano plot, we analyze the impact that small variations in the parameters, relative to their optimal value, have on the value of the error functional *J*_*T*_. The *x*-axis represents the percent variation of the optimal value of the parameter, and the *y*-axis, the variation of the error, normalized by its minimum value, which is the optimal. Therefore, the point (100, 100) is the center and global minimum of all curves in the plot.

Figure 5 shows the sensitivity and stability analysis of the parameters for patient 180. Out of the whole set, the parameter *k*_λ_ shows the more significant variability, which is very small and almost negligible. On the other hand, Figure 6 shows the same analysis for patient 144, which has a very similar glycemic profile, but whose *k*_λ_ is somehow unreliable. Despite sharing almost the same glycemic profile, they have essential differences in their insulinemic trends. The above demonstrates the importance of analyzing both glycemia and insulinemia for having a reliable indicator of the health status of an individual, given that glycemia alone might not be enough. Furthermore, patient 31, who shares little or no properties with the glycemic and insulinemic profile of the other two patients studied above (see Figure 7), has a different sensitivity footprint, proving the relationship between curve shape and parameter reliability.

### Stability of the Solutions in Response to Experimental Errors

**Figure 8:**
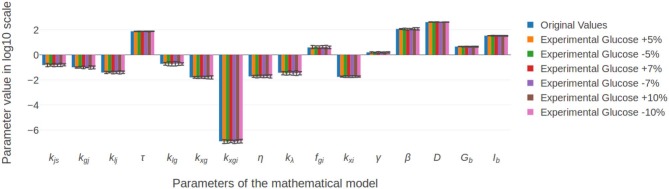
Parameter stability in response to small perturbations in the experimental glucose measurements. Random noise of at most 10% of the reported measurement was added to the original values, and the inverse problem was solved. Results show that, even for the maximum percentage of variation, the difference between the original and final values remained non significant.

After obtaining final values for every parameter, we evaluated their stability in response to small perturbations in the fitted experimental measures. Starting from the experimental OGTT points for a fixed individual (*G*_exp_, *I*_exp_), we simulated a set of virtual patients with OGTT curves (*G*_sim_, *I*_sim_) whose measurements resulted of adding random noise to (*G*_exp_, *I*_exp_), and solved the parameter fitting problem, considering as a starting point the parameters fitted to the original patient. Different amounts of noise were added to the experimental points in all patients. The effects of these variations on all parameter values are shown in Figure 8 for one patient example. All parameters in all generated variations show no significant differences in comparison to the original data, showing that the model allows margins of error associated to both experimental error or time lags at the moment of taking samples without compromising the accuracy of the solution. This invariability is related to the inclusion in the fitting procedure of a component accounting for experimental error, acting as a mathematical buffer, which demonstrates the utility of this approach for individual parameter fitting.

## Conclusions

We presented a new synthetic mathematical model for the G-I dynamics in an Oral Glucose Tolerance Test. Our model only includes parameters with a coherent physiological meaning and, to the best knowledge of the authors, is the first approach which involves Delay Differential Equations (DDE) presented in the literature. The new model can represent radically different G-I behaviors observed in the studied population, including hypoglycemiant individuals, single/double peak patients, and those with practically invariant G-I profiles. In this way, we demonstrate that these unexpected G-I profiles are not experimental errors, but are the result of particular combinations of physiological processes.

We have proposed and numerically implemented a novel strategy for the resolution of the curve-fitting problem, exploiting existing knowledge about the function of the G-I control system, leading to the correct recognition of the individual parameters for the studied cohort of 407 individuals. Our methodology showed to be robust, as the dimensionality of the problem can be dramatically decreased by reshaping the feasible set with the incorporation of an information function and splitting the problem into sequential approaches, thus allowing a correct fitting of the model parameters for each patient. As suggested by the simulations, and afterwards verified by our results, we observed consistency between differences in glycemic and insulinemic OGTT curves and differences in parameter values. The parameters obtained for each and every patient showed to be stable under small perturbations of the experimental measurements, and their sensitivity varied from one patient to another, giving physiological and patient-wise insights of the mechanistic background of the observed trends. This can be asserted particularly by the fact that each parameter in the proposed model represents a unique physiological phenomenon.

Finally, since our model can represent the different forms of control observed so far, to characterize them through parameters with a physiological meaning, and to identify those parameters using a robust methodology for the inverse problem solving, we propose it as a tool for patient evaluation, review of health criteria, and re-definition of clinical normality. Understanding that under the current clinical normality definition there are different ways to achieve glycemic control, parametric analysis of patients would allow the development of individual-oriented treatments, contributing significantly to the preventive and personalized diagnosis of relevant pathophysiological events in the control system. In accordance to this, our future work will include a thorough analysis of parameter values in normal and pathological patients under current definitions and their statistical distributions in broader patient samples, in order to define clinical meaning, normality and pathological criteria for each parameter, which falls out of the scope and length of the present work.

## Data Availability Statement

The datasets generated for this study are available on request to the corresponding author.

## Ethics Statement

The studies involving human participants were reviewed and approved by the Ethics Committee of the San Cristobal Medical Center, Santiago, Chile. The patients/participants provided their written informed consent to participate in this study.

## Author Contributions

SC, DM-O, ÁO-N, and CC: conceptualization, writing, review, and editing. SC, ÁO-N, and CC: methodology. SC and DM-O: validation. SC, DM-O, and ÁO-N: investigation. SC and ÁO-N: supervision. ÁO-N: project administration and funding.

### Conflict of Interest

The authors declare that the research was conducted in the absence of any commercial or financial relationships that could be construed as a potential conflict of interest.
