# Supplementary material for: A Novel Synthetic Model of the Glucose-Insulin System for Patient-Wise Inference of Physiological Parameters From Small-Size OGTT Data
Source: Front Bioeng Biotechnol. 2020 Mar 13;8:195. doi: 10.3389/fbioe.2020.00195 (PMC7083079; doi:10.3389/fbioe.2020.00195)
Supplement: Supplementary file 1 [file Data_Sheet_1.PDF]

Supplementary Material

1 DERIVATION OF HEPATIC-RELATED DIFFERENTIAL MECHANISM

In our analysis of the control of liver glucose production, we decided to keep the model's simplicity without incorporating more state variables. For this reason, we decided not to consider any hepatic internal mechanisms in detail in our model, and we precluded working with the glucagon influence on this phenomenon. It is usually accepted that glucagon starts to play a significant role when glycemia falls below the steady state set point, but glucagon is always secreted in a very low amount. In turn, glucagon stimulates the liver to release glucose into the bloodstream independently from glycemia. Since both pancreatic glucagon production and hepatic glucose release are limited by maximum availability, both processes can be represented by saturable mathematical functions such as the logistic function, the Michaelis-Menten-Monod rectangular hyperbolic functions and the Hill sigmoidal function. All of them are broadly used to represent biological phenomena in which saturation by a maximum availability is present but its exact mechanism is obscure.

With this in mind, we considered that the production of glucose by the liver should be represented by a saturable function which encompasses glucagon production and glucagon-induced glucose release. An additional condition we liked to introduce in our model was to make liver glucose release sensitive to glucose concentration in blood, since glucagon, the initiator of the response, is sensitive to this variable. Moreover, minimum sensitivity to glycemia should be found around the basal glucose value, to ensure control stability and no (dampened) small oscillatory behaviours that are not important during an OGTT. This is a common feature of many biological and non biological control systems, in which the required control reactivity is lowest around the steady state point, in order to softly correct variations in said value with no unstable oscillations. Taking these points into account, we chose G_{prod} to follow a complementary Michaelis-Menten dynamics. This expression allows production of a maximum amount of glucose when there is no glucose in the bloodstream. Glucose release diminishes in an almost linear fashion while glucose levels increase, but slows gradually when glucose values reach a steady state (set point) value. Glucose is always released, although at insignificant rates, if glycemia goes beyond the steady state value. The chosen mathematical expression for this is given by equation S1.

$$G_{\text{prod}} = \frac{k_\lambda}{k_2 + G} \qquad \Longleftrightarrow G_{\text{prod}} = \frac{k_\lambda}{k_2}\left[1 - \frac{G}{k_2 + G}\right]. \tag{S1}$$

To study the differential mechanism behind such expression, we differentiate equation S1, writing G_{prod} as function of itself:

$$\frac{dG_{\text{prod}}}{dG} = -\frac{k_\lambda}{(k_2+G)^2}, \qquad \text{Deriving S1 with respect to G and identifying } G_{\text{prod}},$$

$$\iff \frac{dG_{\text{prod}}}{dG} = \frac{-G_{\text{prod}}}{k_2+G}\cdot\frac{G}{G}, \qquad \text{Multiplying by 1,}$$

$$\iff \frac{dG_{\text{prod}}}{dG} = \frac{-G_{\text{prod}}}{G}\cdot\frac{G+k_2-k_2}{k_2+G}, \qquad \text{Adding zero,}$$

$$\iff \frac{dG_{\text{prod}}}{dG} = \frac{-G_{\text{prod}}}{G}\left(1-\frac{k_2}{k_2+G}\cdot\frac{k_\lambda}{k_\lambda}\right), \qquad ,$$

multiplying by 1 and identifying G_{prod} we may obtain the differential mechanism given by equation S2:

$$\frac{dG_{\text{prod}}}{dG} = \frac{-G_{\text{prod}}}{G}\left(1-\frac{k_2}{k_\lambda}G_{\text{prod}}\right). \tag{S2}$$

We expect the hepatic production to be the responsible to keep up the base glycemic levels in steady state, in the absence of ingested glucose inside the gastrointestinal system. Therefore, by imposing the conditions $G_{\text{prod}} = G_{\text{prod}}(G_b) = G^0_{\text{prod}}$, we have

$$G^0_{\text{prod}} = \frac{k_\lambda}{k_2+G_b} \iff k_2 = \frac{k_\lambda}{G^0_{\text{prod}}} - G_b. \tag{S3}$$

Note that when imposing the steady state condition on equation S1, we obtain equation S4, which is equal to equation 6 in the main manuscript:

$$G_{\text{prod}} = \frac{k_\lambda}{\frac{k_\lambda}{G^0_{\text{prod}}} - G_b + G} \implies G_{\text{prod}} = \frac{k_\lambda}{\frac{k_\lambda}{G^0_{\text{prod}}} + (G-G_b)}. \tag{S4}$$

From the differential mechanism to the expresion of G_{prod}

In the other way around, we may obtain G_{prod} (equation S1) starting from the differential mechanism, only knowing the relationship between k_2 and the other variables in the steady state (equation S3). Calling $\frac{k_2}{k_\lambda} := \alpha$, the differential equation to solve is given by:

$$\frac{dG_{\text{prod}}}{dG} = \frac{-G_{\text{prod}}}{G}\left(1-\alpha G_{\text{prod}}\right). \tag{S5}$$

Knowing that $G_{\text{prod}} \not\equiv 0$ and $G_{\text{prod}} \not\equiv \frac{1}{\alpha}$, we may separate the variables to obtain the following expression

$$\frac{\frac{dG_{\text{prod}}}{dG}}{G_{\text{prod}}\left(1-\alpha G_{\text{prod}}\right)} = \frac{-1}{G}. \tag{S6}$$

Applying partial fraction decomposition on the left side, we can rewrite

$$\frac{1}{G_{\text{prod}}\left(1-\alpha G_{\text{prod}}\right)}=\frac{1}{G_{\text{prod}}}-\frac{-\alpha}{\left(1-\alpha G_{\text{prod}}\right)}, \tag{S7}$$

which facilitates enormously the integration. After the the substitution of equation S7 in equation S6, we may integrate. After proper changes of variables, the integral form of equation S6 is equation S8.

$$\int_{G^0_{\text{prod}}}^{G_{\text{prod}}}\frac{dG'_{\text{prod}}}{G'_{\text{prod}}}-\int_{G^0_{\text{prod}}}^{G_{\text{prod}}}\frac{-\alpha dG'_{\text{prod}}}{\left(1-\alpha G'_{\text{prod}}\right)}=-\int_{G_b}^{G}\frac{dG'}{G'}. \tag{S8}$$

After solving the integrals in S8, we obtain equation

$$\ln\left|\frac{G_{\text{prod}}}{G^0_{\text{prod}}}\right|-\ln\left|\frac{1-\alpha G_{\text{prod}}}{1-\alpha G^0_{\text{prod}}}\right|=-\ln\left|\frac{G}{G_b}\right|, \tag{S9}$$

which can be also expressed as

$$\frac{G_{\text{prod}}\left(1-\alpha G^0_{\text{prod}}\right)}{G^0_{\text{prod}}\left(1-\alpha G_{\text{prod}}\right)}=\frac{G_b}{G} \tag{S10}$$

.

Doing some algebra on equation S10, we may write

$$G_{\text{prod}}\left(1-\alpha G^0_{\text{prod}}+\alpha G_b G^0_{\text{prod}}\right) = \frac{G_b G^0_{\text{prod}}}{G} \tag{S11}$$

$$\Longleftrightarrow \frac{G_{\text{prod}}}{\not{G}}\left[G\left(1-\alpha G^0_{\text{prod}}\right)+\alpha G_b G^0_{\text{prod}}\right] = \frac{G_b G^0_{\text{prod}}}{\not{G}} \tag{S12}$$

We may write α after applying the steady-state conditions S3 as

$$\alpha=\frac{k_2}{k_\lambda}=\frac{1}{G^0_{\text{prod}}}-\frac{G_b}{k_\lambda},$$

or, which is more useful,

$$\alpha G^0_{\text{prod}}=1-\frac{G_b G^0_{\text{prod}}}{k_\lambda}. \tag{S13}$$

Using S13, we may rewrite S12 as

$$G_{\text{prod}}\left[G\left(\cancel{1}-\cancel{1}+\frac{G_bG^0_{\text{prod}}}{k_\lambda}\right)+\left(1-\frac{G_bG^0_{\text{prod}}}{k_\lambda}\right)G_b\right] = G_bG^0_{\text{prod}} \tag{S14}$$

$$\iff G_{\text{prod}}\left[G\frac{G_bG^0_{\text{prod}}}{k_\lambda}-\frac{G_bG^0_{\text{prod}}}{k_\lambda}G_b+G_b\right] = G_bG^0_{\text{prod}} \tag{S15}$$

$$\iff G_{\text{prod}}\frac{\cancel{G_bG^0_{\text{prod}}}}{k_\lambda}\left[(G-G_b)+\frac{k_\lambda}{G^0_{\text{prod}}}\right] = \cancel{G_bG^0_{\text{prod}}} \tag{S16}$$

$$\iff G_{\text{prod}} = \frac{k_\lambda}{\frac{k_\lambda}{G^0_{\text{prod}}}+(G-G_b)}, \tag{S17}$$

which is equivalent to equation S4 and equation 6 in the main manuscript.

2 SUPPLEMENTARY TABLES AND FIGURES

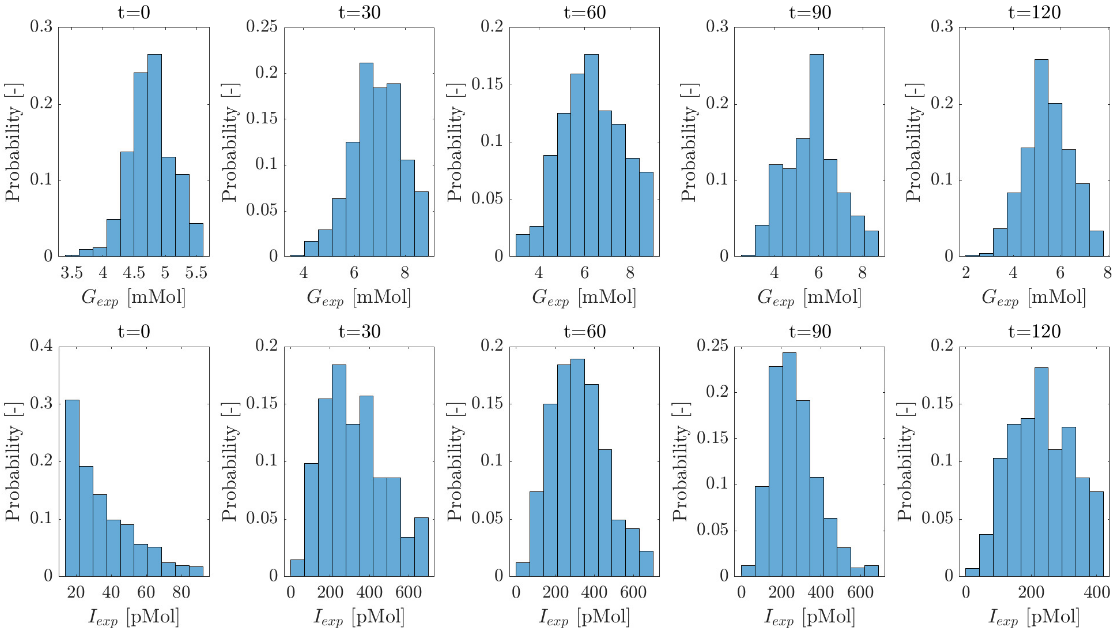

Figure S1: Probability-normalized histogram of experimental measurements of glycemia and insulinemia. Not all the histograms resemble a gaussian profile, therefore the mean and standard deviation might not provide a proper description of them.

Table S1. Statistical summary for blood glucose and insulin measurements from the study cohort of 407 patients

Time [min]	Glycemia [mM]				Insulinemia [pM]			
	Mean	STD	Min. value	Max. value	Mean	STD	Min. value	Max. value
0	4.8	0.4	3.5	5.6	34.7	19.4	14.0	103.0
30	6.8	1.0	3.8	8.9	315.5	154.9	14.0	695.0
60	6.2	1.3	3.0	8.9	310.3	138.8	35.0	695.0
90	5.6	1.2	2.9	8.7	265.6	118.9	45.0	685.0
120	5.3	1.0	2.1	7.7	227.2	95.8	24.0	415.0

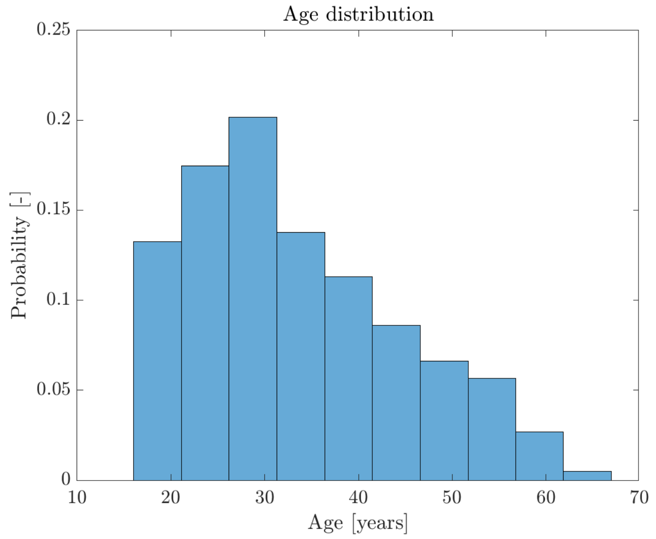

Figure S2: Probability-normalized histogram of age distribution in the cohort.

Table S2. Model Variables

Variable	Units	Physiological meaning	Initial value
S	mmol	Amount of glucose in the stomach	D
J	mmol	Amount of glucose in the jejune	0
L	mmol	Amount of glucose in the ilium	0
G	mM	Blood glucose concentration	G_b
I	mM	Blood insulin concentration	I_b
G_{prod}	mM min^{-1}	Rate of hepatic contribution to glycemia	$G_{\text{prod}}(0)$

Table S3. Model parameters

Parameter	Units	Physiological meaning
k_{js}	min^{-1}	Kinetic constant for stomach emptying
k_{gj}	min^{-1}	Kinetic constant for glucose absorption (jejune)
k_{jl}	min^{-1}	Kinetic constant for glucose delivery from jejune to ilium
τ	min	Time delay between glucose disappearance in jejune and appearance in ilium
k_{gl}	min^{-1}	Kinetic constant for glucose absorption (jejune)
k_{xg}	min^{-1}	Kinetic constant for basal glucose consumption
k_{xgi}	min^{-1}	Kinetic constant for insulin-induced glucose consumption
η	$\mathrm{min}^{-1}\,\mathrm{pM}^{-1}$	Bioavailability of the absorbed glucose
k_{λ}	$\mathrm{mM}^{2}\,\mathrm{min}^{-1}$	Kinetic constant for hepatic glucose release rate
f_{gi}	$\mathrm{min}\,(\mathrm{dm}^{-1})^{3}$	Incretin action conversion factor
k_{xi}	min^{-1}	Kinetic constant for insulin degradation
β	-	Scale for insulin production saturation
γ	-	Scale for insulin production acceleration
